# Salt-responsive bermudagrass microRNAs and insights into light reaction photosynthetic performance

**DOI:** 10.3389/fpls.2023.1141295

**Published:** 2023-02-15

**Authors:** Shugao Fan, Erick Amombo, Sheila Avoga, Yating Li, Yanling Yin

**Affiliations:** ^1^ School of Resources and Environmental Engineering, Ludong University, Yantai, China; ^2^ African Sustainable Agriculture Institute, Mohammed VI Polytechnic University, Laayoune, Morocco; ^3^ Key Laboratory of Plant Germplasm Enhancement and Specialty Agriculture, Wuhan Botanical Garden, Chinese Academy of Science, Wuhan, China

**Keywords:** electron transport, microRNA, salt stress, bermudagrass, target gene

## Abstract

**Introduction:**

Bermudagrass (*Cynodon dactylon* L.) is a warm-season grass with high drought and salt tolerance. However, its cultivation as a silage crop is limited by its lower forage value when compared to other C4 crops. Because of its high genetic variability in abiotic stress tolerance, bermudagrass-mediated genetic breeding offers significant promise for introducing alternative fodder crops in saline and drought-affected regions, and improved photosynthetic capacity is one way for increasing forage yield.

**Methods:**

Here, we used RNA sequencing to profile miRNAs in two bermudagrass genotypes with contrasting salt tolerance growing under saline conditions.

**Results:**

Putatively, 536 miRNA variants were salt-inducible, with the majority being downregulated in salt-tolerant vs sensitive varieties. Also, seven miRNAs putatively targeted 6 genes which were significantly annotated to light reaction photosynthesis. Among the microRNAs, highly abundant miRNA171f in the salt tolerant regime targeted Pentatricopeptide repeat-containing protein and dehydrogenase family 3 member F1 both annotated to electron transport and Light harvesting protein complex 1 genes annotated to light photosynthetic reaction in salt tolerant regime vs salt sensitive counterparts. To facilitate genetic breeding for photosynthetic capacity, we overexpressed miR171f in *Medicago tracantula* which resulted in a substantial increase in the chlorophyll transient curve, electron transport rate, quantum yield of photosystem II non photochemical quenching, NADPH and biomass accumulation under saline conditions while its targets were downregulated. At ambient light level the electron transport was negatively correlated with all parameters while the NADPH was positively associated higher dry matter in mutants.

**Discussion:**

These results demonstrate that miR171f improves photosynthetic performance and dry matter accumulation via transcriptional repression of genes in the electron transport pathway under saline conditions and thus a target for breeding.

## 1 Introduction

Soil and water salinity are serious abiotic factors globally (Cheeseman, 1988; Allakhverdiev et al., 2000). Other than natural causes, soil salinity has been aggravated by excessive use of saline water for irrigation (Shrivastava and Kumar, 2015). As a result, soil structure and water-holding capacity have deteriorated dramatically over the years (Yadav et al., 2011). Soil salinity is mostly constituted by NaCl due to its remarkable solubility in water (Tavakkoli et al., 2010). Excessive buildup of Na^+^ and Cl^-^ ions are phytotoxic and imposes physiological drought (Hasegawa et al., 2000; Munns, 2002). Therefore, plants growing in these soils must adapt to survive. This poses a serious threat to plant growth and crop productivity, especially in arid and semi-arid regions where drought is already a major challenge (Alori et al., 2020). Understanding soil salinity *in toto* is a complex process. This is attributed to the soil’s highly heterogeneous nature of salt occurrence making it hard to develop a regional level policy (Hassani et al., 2021). Concentrating on the various bioprocesses that plants demonstrate to survive salt stress at genotypic and phenotypic levels is therefore more viable.

Genetic breeding and cultivating high-yielding forages in saline areas are ideal for addressing food insecurity through livestock production (Fita et al., 2015). The photosynthetic pathway is one of the most reliable indicators of plant performance and productivity and thus a target for breeding (Ashraf and Harris, 2013). For example, photosynthesis is the primary driver of biomass accumulation, and the efficiency with which a crop transforms CO_2_ into metabolites is a crucial determinant of eventual crop yield (Simkin et al., 2019). Also, other than being reliable abiotic stress tolerance level indicators (Guidi et al., 2019), the slow induction of chlorophyll *a* fluorescence kinetics variable such as the maximum quantum yield of photosystem II, the electron transport rate, nonphotochemical quenching, and the photosynthetic performance index have been used as targets for forward and reverse genetic breeding for efficient yield gain (Sherstneva et al., 2022). Thus, increased salt tolerance and forage yields can be achieved by maintaining a balance of light use efficiency and photosynthetic carbon uptake and assimilation efficiency (Busch et al., 2018).

Bermudagrass (*Cynodon dactylon* L.) is a warm-season grass with excellent saline-land potential (Chen et al., 2019). Despite the incredible potential of photosynthetic efficiency of light conversion to harvestable biomass in bermudagrass, it has yet to be extensively examined due to a scarcity of genetic information for candidate gene discovery. As a result, bermudagrass remains to be a potentially rich genetic resource for breeding alternative forages for salt tolerance.

Small RNAs, which influence the transcription of their targets *via* RNA-RNA interactions, are one of the most remarkable regulatory molecules (Helge and Witold, 2008), with microRNAs being one of the best-understood groups. MicroRNAs influence gene expression *via* transcriptional repression (Voinnet, 2009). Llave et al. (2002); Park et al. (2002) and Reinhart et al. (2002) rigorously described miRNA and their targets in Arabidopsis which opened doors for subsequent studies. Many miRNAs have since been implicated with salt tolerance in various grass family members, including switchgrass (*Panicum virgatum*) (Xie et al., 2013) and wild emmer wheat (*Triticum turgidum*) (Feng et al., 2017).

While miRNAs’ potential roles in photosynthesis have been elucidated in Arabidopsis, tobacco, and rice (Zhang et al., 2017; Pan et al., 2018), studies that comprehensively focus on photosynthetic regulation by miRNAs under salt stress are scarce. Closing this gap will be critical in availing a genomic platform on the photosynthetic response of bermudagrass to salt stress and identifying potential targets for breeding for photosynthetic performance under saline conditions. Here, we did comparative research in two bermudagrass cultivars with differing salt tolerance levels to investigate the regulatory network of miRNA, their targets in photosynthesis, and their potential function in plant breeding.

## 2 Materials and methods

Here, plant resources comprised two bermudagrass varieties, cultivar 43(C43, salt tolerant) and cultivar 198 (C198, salt sensitive), collected from the germplasm center of the Wuhan Botanical Garden, Chinese Academy of Science. We screened for salt tolerance in our previous study (Hu et al., 2015). Freshly harvested stolons with two internodes were planted in plastic pots containing soil mixed with sand (ratio of 2:1, v/v) in the greenhouse. After 14 days of growth and maturation, the plants were transplanted into plastic pots (7 cm in diameter and 9 cm in depth) filled with coarse silica aggregate as the plant anchor medium. The pots were hung in rectangular containers filled with half-strength Hoagland’s solution, replenished three times a week at one-day intervals, and refreshed weekly. For 14 hours, plants were cultivated in a greenhouse with a temperature regime of 25°C at night and 28°C during the day, with photosynthetically active radiation levels of 800 mol m^-2^ s^-1^. After 21 days, the half-strength Hoagland’s solution was added with 200 mM NaCl, and after 7 days of salt treatment, 0.5 g of fresh leaves were excised and instantly frozen in liquid nitrogen. Samples were collected from multiple pots from each treatment for RNA sequencing (three repetitions), quantitative real-time PCR (real-time qPCR) validation (three repeats), and physiological measurements (four biological replicates for each physiological parameter) all at the same time.

### 2.1 RNA extraction, sequencing, and small RNA library construction and data processing

Total RNA was extracted using Trizol reagent (Invitrogen, CA, USA) according to the manufacturer’s instructions. The amount and purity of RNA were determined using the Bioanalyzer 2100 and the RNA 6000 Nano LabChip Kit (Agilent, CA, USA). The experiment was carried out in line with Illumina Incorporation’s standard methods, which included library preparation and sequencing studies. The TruSeq Small RNA Sample Prep Kits were used to construct small RNA sequencing libraries (Illumina, San Diego, USA). The characteristics of the miRNA were applied after the total RNA was extracted from the sample. RNA ligase enzyme was used to attach single-stranded DNA 3’ and 5’ sticky ends to short RNAs in sequence. The short RNA sequence with the connected ends was reverse-transcribed using primers complementary to the three ends, and the resulting cDNA sequence was PCR-amplified. Finally, the PCR product of 140-160 base pairs (bp) in length was recovered using a 6% polyacrylamide Tris-borate-EDTA gel to conclude the library preparation. Illumina Hiseq2000/2500 was used to sequence the library that was created. The data quality of the sequence was assessed using Illumina FastQC software (Ichikawa Biosystems ACGT101-miR LC Sciences, Houston, Texas, USA), which was created separately by the firm. Sequencing junctions, unusual miRNA sequences, and incomplete sequences were removed from the analysis. Following that, base lengths of 18 and >25 nucleotides (nt) were screened, as well as miRNA-free sequences such as mRNA, RFam (containing rRNA, tRNA, snRNA, snoRNA, and so on), and Repbase (www.girinst.org).

### 2.2 Detection of known and novel miRNAs

Following the cleanup and removal of the remaining sequences, Bowtie2 (Langmead and Salzberg, 2012) was used to align the precursors of specific species in the miRBase database. Variations in length at the 5’ and 3’ ends and a mismatch within the sequence were permitted in the alignment analysis. The miRNAs that were matched to the mature sequence component of the given species were categorized as known miRNAs. If the identified sequence could not be linked to the corresponding site of a known miRNA, it was designated a novel 5p or 3p miRNA candidate sequence. The new mismatched sequences were again processed through a Bowtie alignment to precursors of other chosen species in miRBase, and the aligned miRNA precursor sequences were recognized as unique. The RNA secondary structures were predicted using RNAfold soft (http://rna.tbi.univie.ac.at/cgi-bin/RNAfold.cgi).

### 2.3 Analysis of differentially expressed miRNAs and prediction of their targets

The expression levels of differently expressed miRNAs in the four regimes were examined using the Fisher exact test. The fold changes of miRNA readings were calculated as the ratio of salt treatment over corresponding control for each cultivar, as well as salt-tolerant C43 salt vs salt-sensitive C198 salt. The fold change and P-value were used to determine the significance of the expression (significant *absolute fold change 1 and P-value 0.05; highly significant **, absolute fold change 1 and P-value 0.01). To predict DEM target genes, we utilized the website service psRNATarget (http://plantgrn.noble.org/psRNATarget/?function=3) in conjunction with our prior bermudagrass EST data (Hu et al., 2015). The gene ontology (GO) keywords and KEGG Pathway of the miRNA targets were also annotated to diverse functions.

### 2.4 Expression of photosynthesis-related miRNAs and their targets by real-time qPCR

To identify and validate the differentially expressed miRNAs in the photosynthesis pathway, six miRNAs with high expression levels (at least 1000) in all libraries were selected from conserved families (miR171f, miR319, MiR156a, and miR159), and non-conserved (MIR305714, MIR46018, and MIR2836) and amplified using real-time qPCR to examine their expression. The forward miRNAs primers were designed based on the full miRNAs sequence, while the reverse primer was the universal reverse primer (5’GTGCAGGGTCCGAGGT3’). The stem-loop primer, used for miRNA cDNA synthesis, was designed according to Chen et al. (2005). The 18S rRNA was taken as the reference gene. Similarly, the expression profiles of the respective miRNA target genes were assayed by real-time qPCR. In summary, a 10-µg aliquot of RNA was used for the RT reaction followed by the addition of oligo(dT) primer. The RT reaction was performed by MMLV reverse transcriptase (Toyobo, Osaka, Japan) according to the supplier’s manual. For real-time qPCR, *ACTIN* for bermudagrass was used as a housekeeping gene as the internal control. To calculate the relative expression, the 2^-ΔΔCt^ method was used and Student’s *t*-test was performed to compare differences in expression profile. The means were considered significantly different when P ≤ 0.05.

### 2.5 Heterologous overexpression of miR171f

After confirming that miR171f responds to salt stress, we generated transgenic *Medicago* constitutively expressing miR171f. The constitutive expression construct of miR171f was introduced into wild-type (WT) plants *via Agrobacterium tumefaciens*-mediated transformation. The selectable marker gene, Hyg conferring hygromycin resistance was amplified from the genomic DNA of regenerated plants and controls. The expression levels of pre-miR171f and mature miR171f were then compared between the wildtype and transgenic plants to determine whether the bermudagrass pre-miR171f was successfully integrated into the genome of *Medicago*, transcribed, and properly processed.

### 2.6 Chlorophyll a fluorescence and NADPH activity

After dark adaptation, the maximum chlorophyll fluorescence (F_M_) was determined every 30s by saturation pulse (800 ms, 2,700 μmol quanta m^-2^·s^-1^). Light intensity-dependent parameters of _Φ_PSII, ETR, and NPQ, were measured with a range of intensities from 0, 200, 400, 600, 800, to 1000 μmol·m^-2^·s^-1^ and calculated. Measurement of photosynthetic light response curves was performed on a single fully expanded leaf exposed to a light source. All the measurements were conducted using the Li-6400 portable photosynthesis system. The NADPH activity was determined by the reduction of the tetrazolium salt XTT by O^2-^ following Kaundal et al. (2012) protocol. In the presence of O^2-^, XTT generated a soluble yellow formazan that can be quantified spectrophotometrically.

## 3 Results

### 3.1 Identification of known and novel miRNAs

In the C43 CK, C43 salt, C198 CK, and C198 salt libraries, there were 10,945,629, 9,755,632, 12,520,523, and 19,469,714 raw reads as well as 2,775,967, 3,047,166, 2,804,775, and 3,647,171 unique reads. After excluding non-coding RNA groups such as rRNAs, tRNAs, snoRNAs, snRNAs, and other contaminants, as well as those with a length of 18nt and >25 nt, a total of 4,897,423, 4,464,136, 4,670,166, and 4,852,132 valid reads, as well as 1,833,027, 1,855,458, 1,320,178, and 1,556,539 unique reads were obtained (**Table 1**).

**Table 1:** Small RNA reads from sequencing of four bermudagrass libraries.

	C198_ck		C198_salt		C43_ck		C43_salt	
Type*	Total	unique	Total	unique	Total	unique	Total	unique
Raw reads	582,201	118,587	349311	80,185	208,395	45,208	186,778	55,831
3ADT&length filter	637,930	138,105	427479	102,454	286,206	68,519	209,685	70,560
Junk reads	681,016	155,326	532807	136,668	471,632	108,808	285,641	105,371
Rfam	1,120,506	262,989	1116018	321,331	1,091,790	338,433	973,513	360,002
Repeats	656,616	230,351	935309	356,690	1,031,775	420,110	1,329,076	514,502
valid reads	421,789	144,721	561401	187,692	609,651	235,709	541,705	267,458
rRNA	407,452	217,025	589133	269,087	860,842	506,969	624,339	376,593
tRNA	162,656	53,074	340674	102,432	337,132	109,271	313,399	105,141
miRNA	4,670,166	1,320,178	4852132	1,556,539	4,897,423	1,833,027	4,464,136	1,855,458

**Figure 1:**
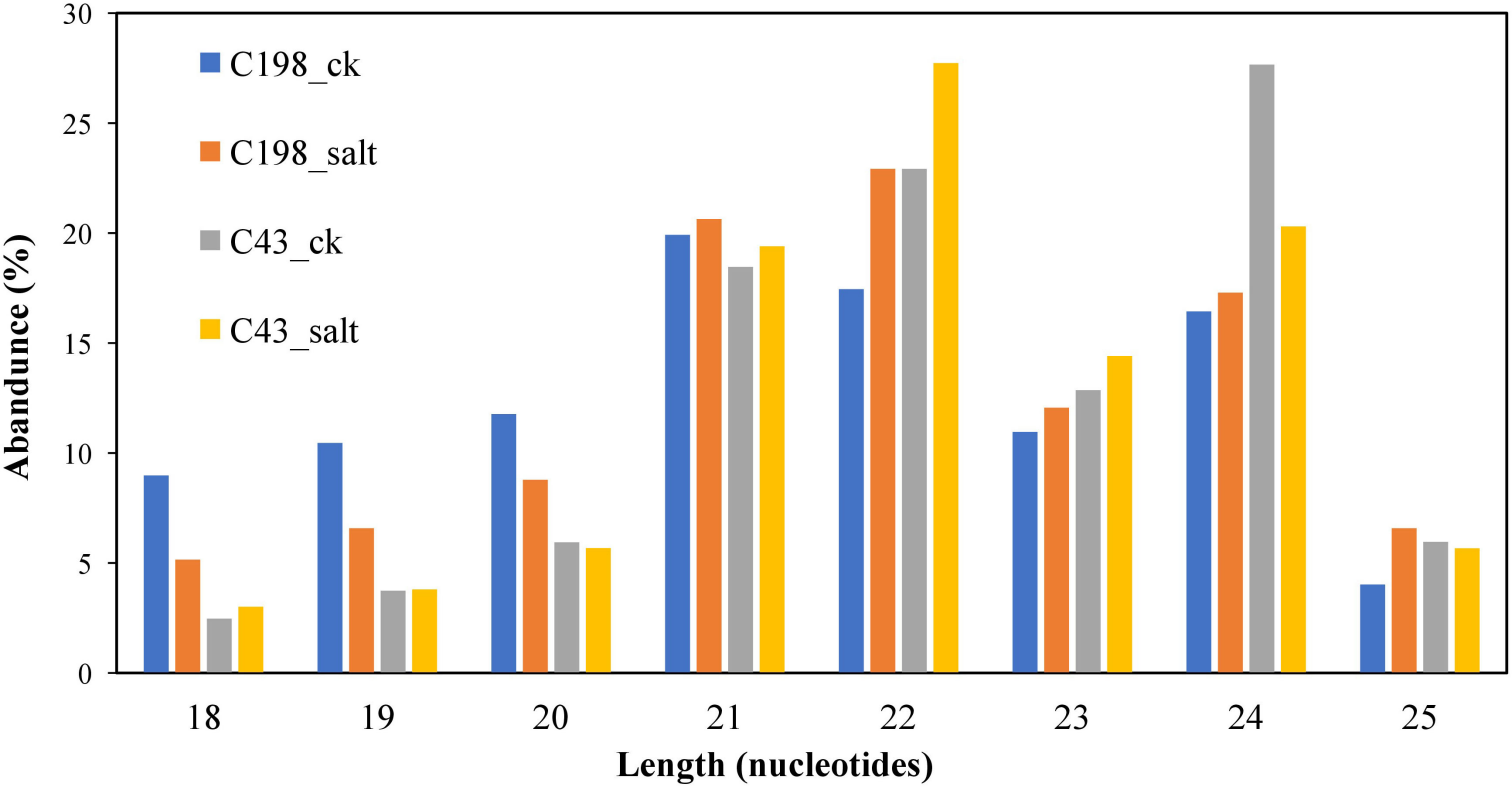
Length distribution of unique sequences after cleaning.

After length filtering, most of the length data lay between 20 and 24 nt. C198 ck was dominated by 21 nt (19.92%) in salt-sensitive libraries, whereas C198 salt was dominated by 22 nt (22.29%). C43 ck was dominated by 24 nt (27.66%), whereas C43 salt was dominated by 22 nt (20.30%). The 18 nt had the least miRNAs in all the libraries (**Figure 1**; **Table S1**).

**Figure 2:**
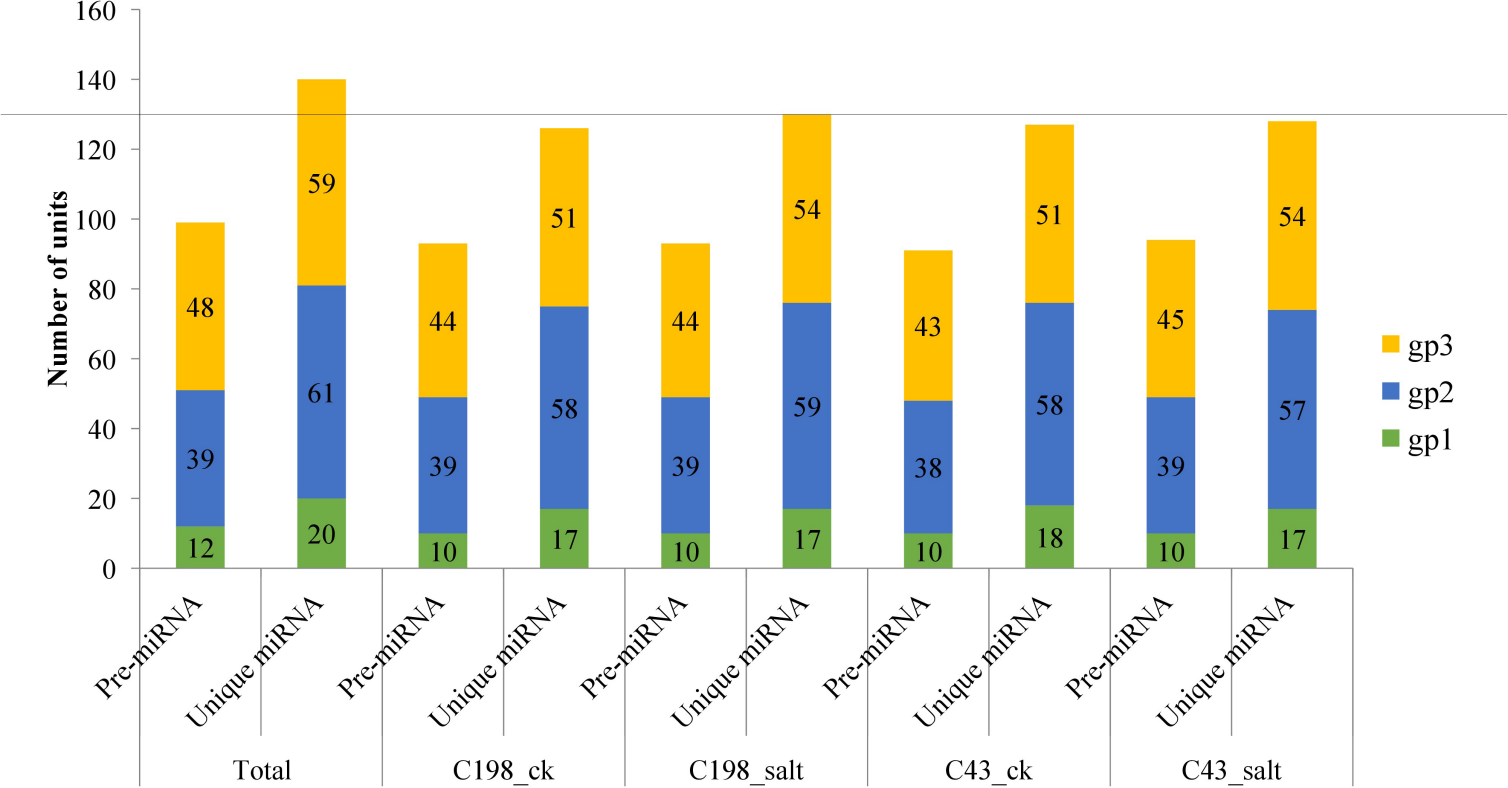
Plot showing the characterization of predicted vs mature microRNAs (a) group 1 (b) group 2 and (c) group 3. Group 1 represents sequences that were mapped to miRNAs/pre-miRNAs of specific species in miRbase as well as bermudagrass EST; group 2 represents sequences that were mapped to miRNAs/pre-miRNAs of selected species in miRbase, and the mapped pre-miRNAs were not further mapped to genome/EST. group 3 represented the reads that were not mapped to pre-miRNAs of selected species in miRbase but were rather mapped to genome/EST.

After matching the identified miRNAs with miRbase, we classified them into three groups: gp1, gp2, and gp3. Gp1 was made up of 20 mature miRNAs that were mapped to specific species’ miRNAs/pre-miRNAs, and the pre-miRNAs were then mapped to our bermudagrass EST. Gp2 consisted of 61 mature reads that were mapped to miRNAs/pre-miRNAs of select species in miRbase but were not further mapped to bermudagrass EST. As a result, the known miRNAs were established by these two groups (gp1 and gp2). Gp3 had 59 mature miRNAs that were not linked to specific species miRNAs in miRbase, however, the readings were mapped to our bermudagrass EST (**Figure 2**; **Table S2**).

**Figure 3:**
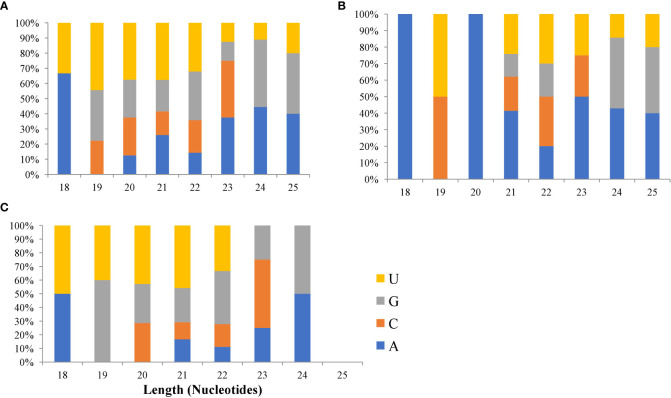
Plots of first nucleotide bias of **(A)** group 3 **(B)** group 2 and **(C)** group 1 microRNAs. The upper plot represents the first nucleotide. Gp1 represents sequences that were mapped to miRNAs/pre-miRNAs of specific species in miRbase as well as bermudagrass EST; group 2 represents sequences that were mapped to miRNAs/pre-miRNAs of selected species in miRbase, and the mapped pre-miRNAs were not further mapped to genome/EST. group 3 represented the reads that were not mapped to pre-miRNAs of selected species in miRbase but were rather mapped to genome/EST.

As a result, we considered these to be novel sequences. The sequences were thus labeled as p3/p5 to distinguish them from the previously published sequences. The sequences were further probed to match Hofacker’s prediction requirements for novel miRNAs (Hofacker, 2003). As a result, 59 had very low abundance with dissociation energy ranging from -32.3 to -142.30 kcal mol^−1^ which is consistent with the degradation principle of miRNA during the early generation process. Their GC content ranged from 30-81%, indicating that the novel miRNAs were stable. The minimal free energy index (MFE) ranged from 0.8 to 1.5 which is higher than that of other small noncoding RNAs reported by Zhang et al. (2018). In addition, uracil (U) and adenine (A) were the dominant nucleotides in the first positions of the miRNAs (**Figures 3A–C**; **Table S3**) which is not only a characteristic of mature miRNA but also plays a vital role in the miRNA-target interaction.

### 3.2 Conservation profile of detected miRNA

**Figure 4:**
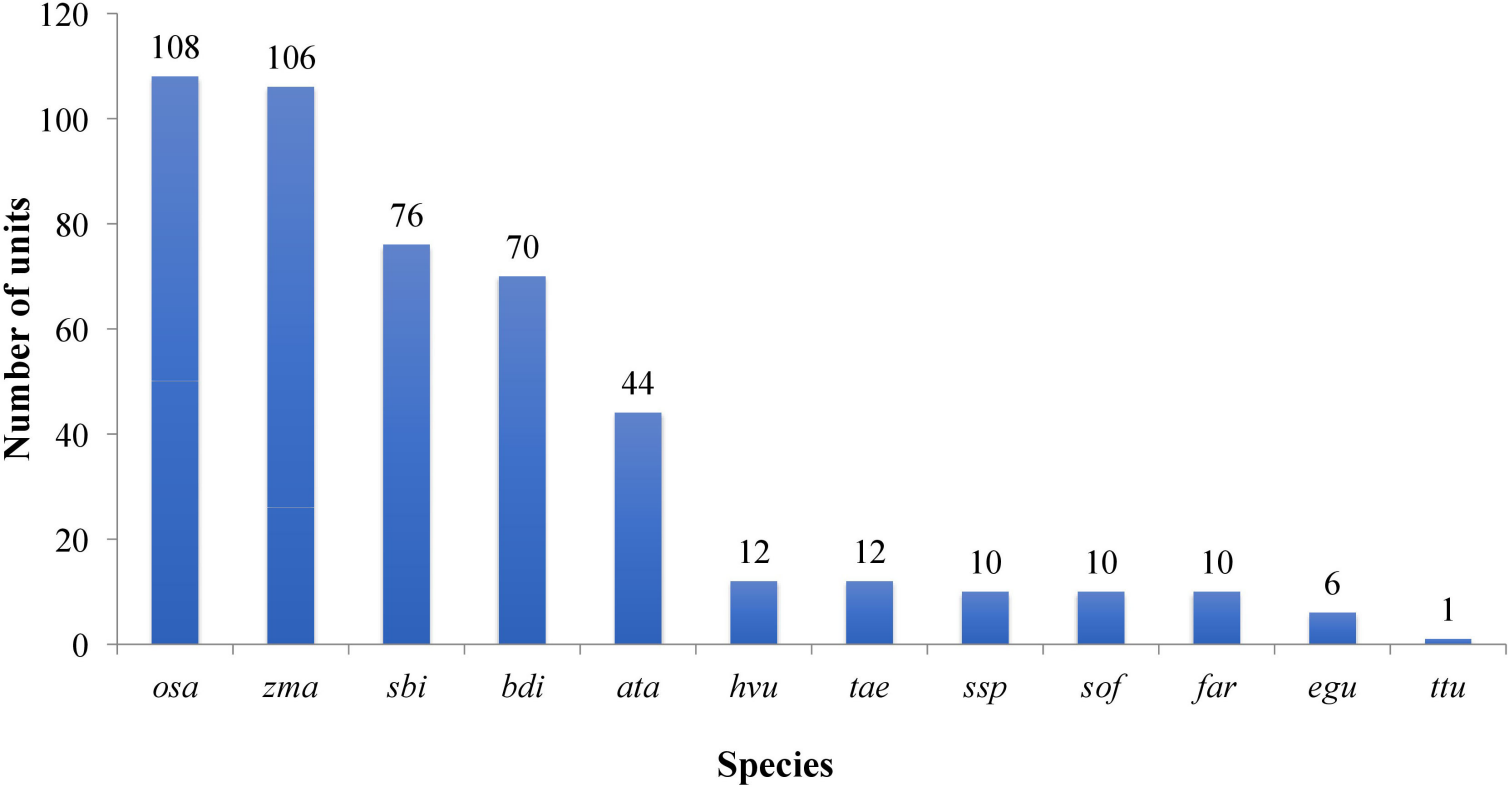
Plot showing the conservation profile of identified miRNAs. Ata- *Aegilops tauschii*, bdi- *Brachypodium distachyon*, egu- *Elaeis guineensis*, far- *Fragaria ananassa*, Hvu- *Hordeum vulgare*, osa-*Oryza sativa*, sbi- *Sorghum bicolor*, sof- *Saccharum officinarum*, Tae-*Triticum aestivum*- ssp- *Saccharum sp*, zma-*Zea mays*, ttu-*Triticum turgidum*.

All the known miRNAs were shown as variants, which were defined as ‘isomiRs. For example, R-n means that the detected miRNA sequence was n base/s less than the known representative miRNA sequence in miRbase (rep_miRSeq) in the right side; L+n means the detected miRNA sequence is n base more than known rep_miRSeq in the left side; R+n means the detected miRNA sequence is n base more than known rep_miRSeq on the right side; 2ss5TC13TA means 2 substitutions (ss), which are T≥C at position 5 and T≥A at position 13. If there was no matching annotation, the detected miRNA sequence is the same as known rep_miRSeq. Among the known group, miRNAs belonged to 16 conserved families while the rest belonged to 13 non-conserved families (**Figure 4**; **Table S4**).

### 3.3 Identification of differentially expressed miRNAs

**Figure 5:**
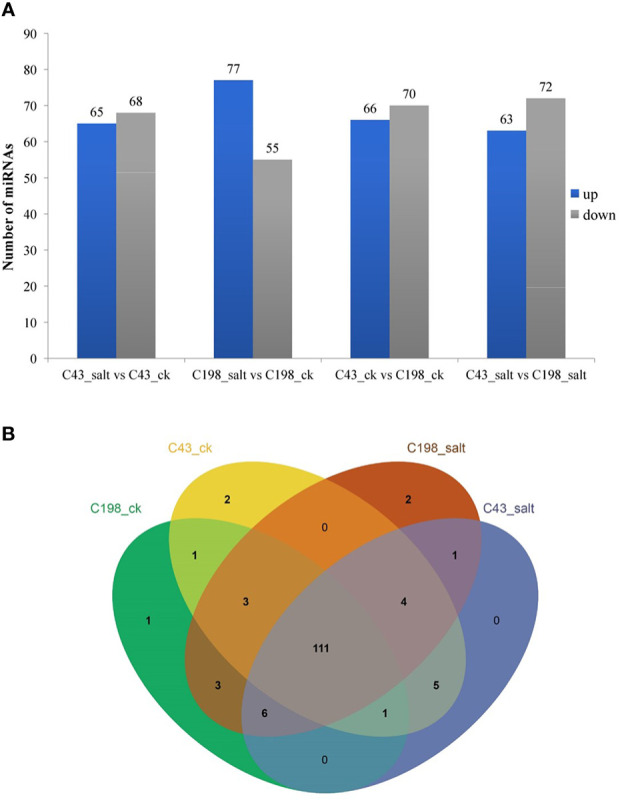
**(A)** Bar plot of differentially expressed miRNAs and their expression level. **(B)** Venn diagram showing miRNA sharing in four bermudagrass libraries with contrasting salt tolerance levels.

To identify differentially expressed miRNAs (DEM) under salinity stress, the analysis of differential expression patterns was performed by statistical comparison between four libraries. In each library, the expression level of mature miRNAs was normalized to Transcripts Per Kilobase Million (TPM) and compared between salt treatment vs control as well as salt-tolerant vs salt-sensitive cultivars. Totally, 536 miRNAs were expressed in the four libraries. Among them, 111 miRNAs were shared among all four libraries. In addition, in the C43 vs C198 salt regime, the total DEM belonged to 12 conserved families i.e., miR160, miR164, miR166, miR167, miR156, osa-miR171, osa-miR172, miR390, miR396, miR162, miR444 and miR393, 6 non-conserved families i.e., miR812, miR6255, miR818, miR2118, miR9482, and miR5384, while 57 were novel and most of the DEM were downregulated (72 downregulated and 63 upregulated) (**Figure 5A**; **Table S5**). Among the novel miRNAs, the expression abundance was very low, for instance, only 11 miRNAs had an expression abundance of more than 10 reads. Out of these relatively abundant novel miRNAs, 6 were downregulated while five were upregulated under salt stress. Cluster analysis revealed a total of 7 miRNA clusters in the bermudagrass. Cluster 2 constituted the largest number of miRNA variants i.e., MIR444c, miR444a, MIR444d, miR444c, and MIR444c. Some pre-miRNAs in the same cluster had similar expression patterns with their mature counterparts, for example in cluster 6 MIR159d was not only located in the same genomic locus with miR159c but were all downregulated in both the control and salt regimes of C43 vs C198 (**Table S6**). A total of 111 microRNAs were shared among all the four libraries. Exclusive microRNAs were 1, 2, 2, and 0 in ck198, ck43, salt 198 and salt 43 respectively (**Figure 5B**).

### 3.4 miRNA targeting salt-tolerant and photosynthesis-related genes

**Figure 6:**
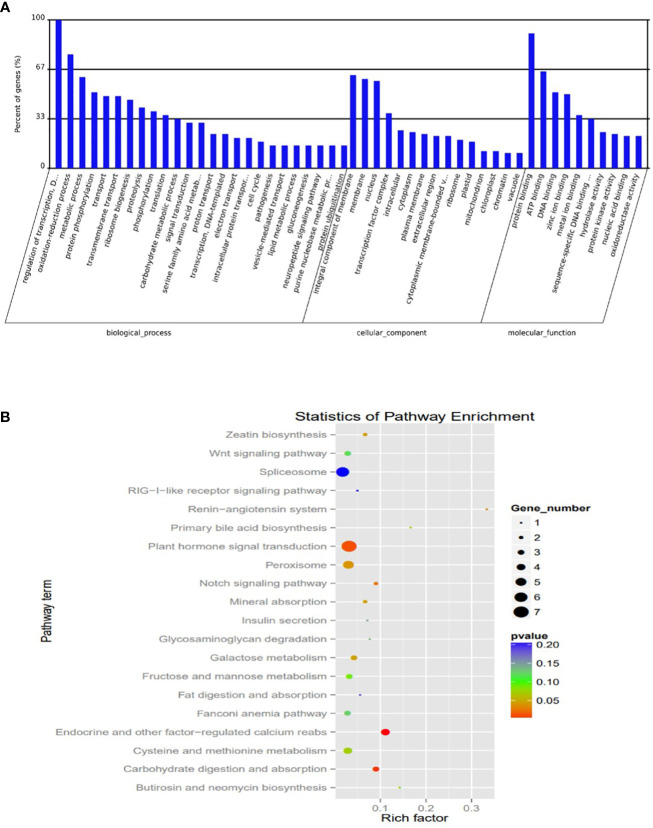
Plots of Characterization was based on **(A)** Gene ontology (GO) categories in the root apex of four bermudagrass libraries **(B)** Kyoto Encyclopedia of Genes and Genomes (KEGG) pathway enrichment.

To understand the potential transcriptional roles of the DEM, it was crucial to focus on their targets in salt tolerant vs sensitive regimes. Putative 1891 genes were targeted by 139 mature miRNAs. The number of targets per miRNA ranged from 1 to 287. Notably, microRNAs targeting most genes are novel with very low abundance. Also, many miRNAs targeted a single gene (**Table S7**). To understand the potential function of the miRNA targets, we integrated their gene functions in the GO database with previously published literature. A total of 584 target genes were annotated to various GO terms which were classified into three major categories i.e., ‘molecular function’, ‘cellular component’, and ‘biological process’ (**Figure 6A**; **Table S8**).

The KEGG analysis revealed the top 20 enriched pathways which were identified with 55 genes “plant hormone signal transduction” was the most significantly enriched term with respect to the richness factor and gene number (**Figure 6B**; **Table S9**). This suggested that most of the targets in this category might play an important role in hormonal salt stress signal perception and transduction by roots of bermudagrass.

**Figure 7:**
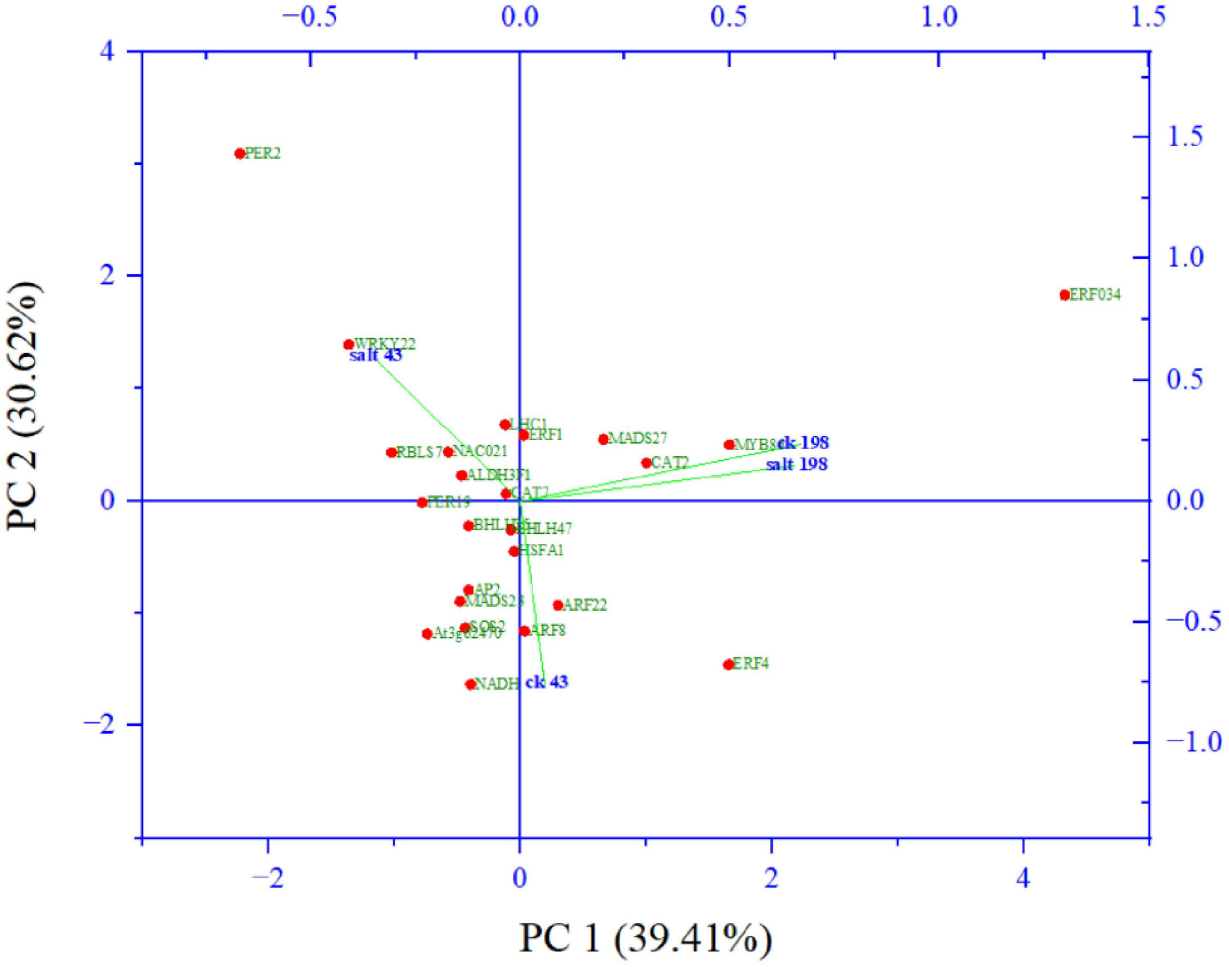
Principal component analysis of abundant salt responsive and photosynthesis target genes from gene ontologies.

Among them key salt tolerance pathways are significantly enriched such as the transcription factor complex. Under this category transcription factors from major salt-tolerant families such as NAC, WRKY, ERF, bHLH, MADs, and SOS, are enriched. Component 2 which accounted for 31% of the variation composed of members salt tolerant genes including WRKY22 (Li et al., 2018), NAC021 (Yang et al., 2020), CAT7 (Karam et al., 2016) and two photosynthesis genes *light harvesting complex 1* (Yang et al., 2019) and *aldehyde dehydrogenase 1* (**Figure 7**).

To study the role of miRNA in photosynthesis, effectively, we filtered highly abundant miRNAs whose targets were assigned to relevant gene ontologies and three conserved miRNAs miR171f, miR319a and miR159c were highly abundant. The most abundant is miR171f targeting *Pentatricopeptide repeat-containing protein At3g62470* which is significantly enriched to a biological function ‘electron transporter, transferring electrons within the cyclic electron transport pathway of photosynthesis activity. miR319a and miR159c each target *NAD-dependent protein deacetylase SRT1* which are significantly enriched to the biological function *‘*NAD^+^ binding’. The rest are non-conserved miRNAs MIR305714, MIR46018, MIR2836, and MIR305714, three targeting genes annotated to electron carrier activity (MIR305714, MIR46018, MIR2836) and MIR305714 targeting protein *SCARECROW SCR* significantly annotated to the stomatal complex morphogenesis (**Table 2**).

**Table 2:** Target gene ontologies for highly downregulated microRNAs related to photosynthesis.

microRNA	ID	Gene target annotation	Abundance	Gene ID	GO term
miR156a	comp89548_c0	Premnaspirodiene oxygenase OS=Hyoscyamus muticus GN=*CYP71D55*	115	GO:0009055	electron carrier activity
MIR46018	comp105422_c0	Uncharacterized protein At5g39865 OS=*Arabidopsis thaliana* GN=*At5g39865*	763	GO:0009055	electron carrier activity
miR171f	comp116117_c0	Aldehyde dehydrogenase family 3 member F1 OS=*Arabidopsis thaliana* GN=*ALDH3F1*	1104	GO:0006118	electron transport
miR171f	comp124273_c0	“Pentatricopeptide repeat-containing protein At3g62470, mitochondrial OS=*Arabidopsis thaliana* 3g62470	3172	GO:0045156	electron transporter, transferring electrons within the cyclic electron transport pathway of photosynthesis activity
miR171f	comp121036_c0	Light harvesting protein complex1 OS=*Oryza sativa* subsp. indica GN= *LHC1*	1753	GO:0070403	Light harvesting protein complex
miR159c	comp121036_c0	NAD-dependent protein deacetylase *SRT1* OS=*Oryza sativa* subsp. indica GN=*SRT1*	1755	GO:0070403	NAD+ binding
MIR305714	comp122900_c0	Protein SCARECROW OS=*Zea mays* GN=*SCR*	505	GO:0010103	stomatal complex morphogenesis

**Figure 8:**
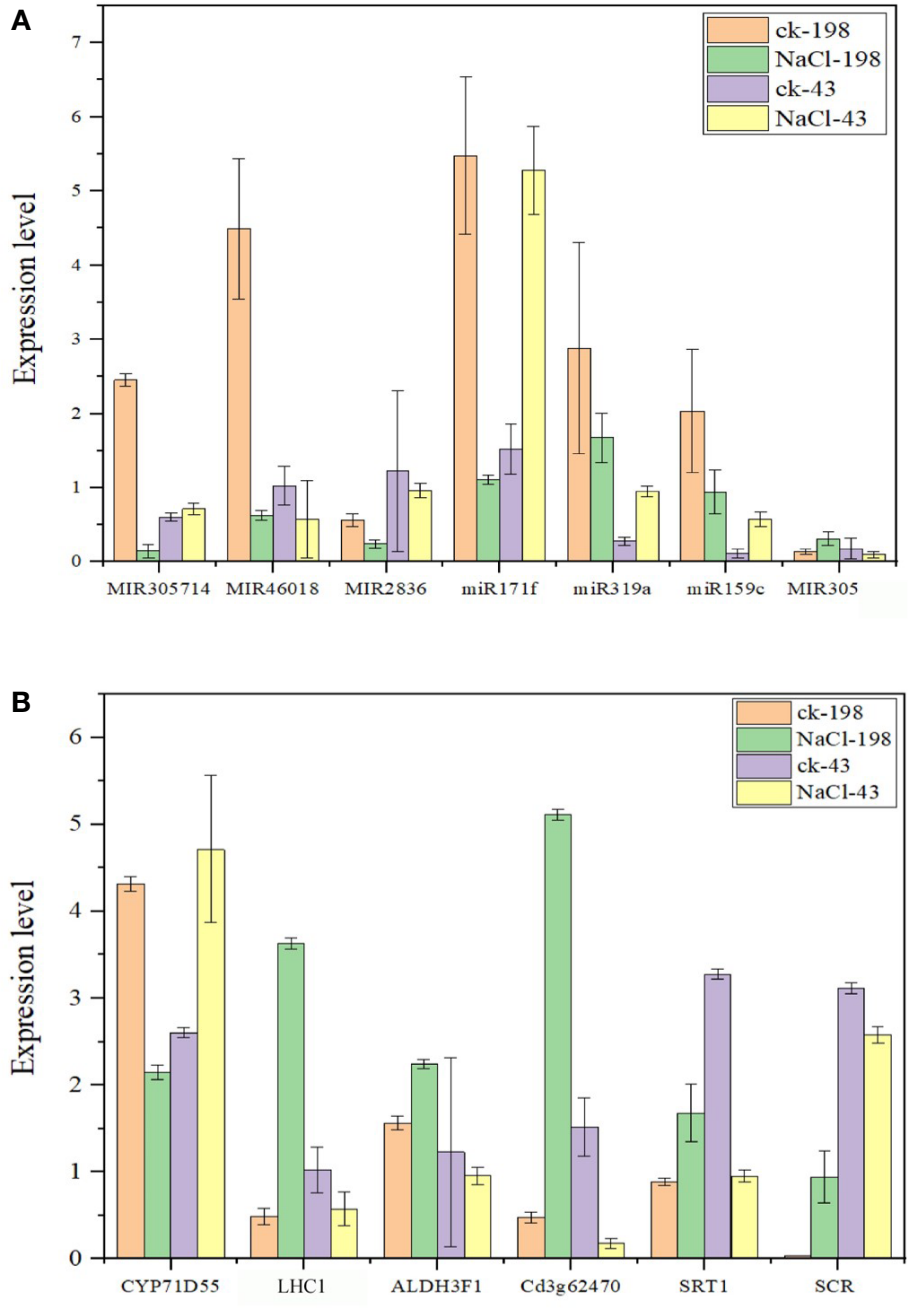
Bar plots showing real-time qPCR validation of miRNAs with high expression levels from sequencing data (at least 1000 in salt tolerant vs sensitive regime) **(A)** and respective target genes for the selected miRNAs **(B)**. The data are the means of four replicates. Fold change below 1 is downregulation and above 1 is upregulation.

To proceed with these miRNAs, we validated the expression *via* real-time PCR using primers (**Table S10**). All the miRNAs are downregulated in sequencing and PCR analysis except miR171f whose target is downregulated. Therefore, we considered miR171f a target for forward genetic functional analysis (**Figures 8A, B**).

### 3.5 Heterologous MiR171f overexpression

**Figure 9:**
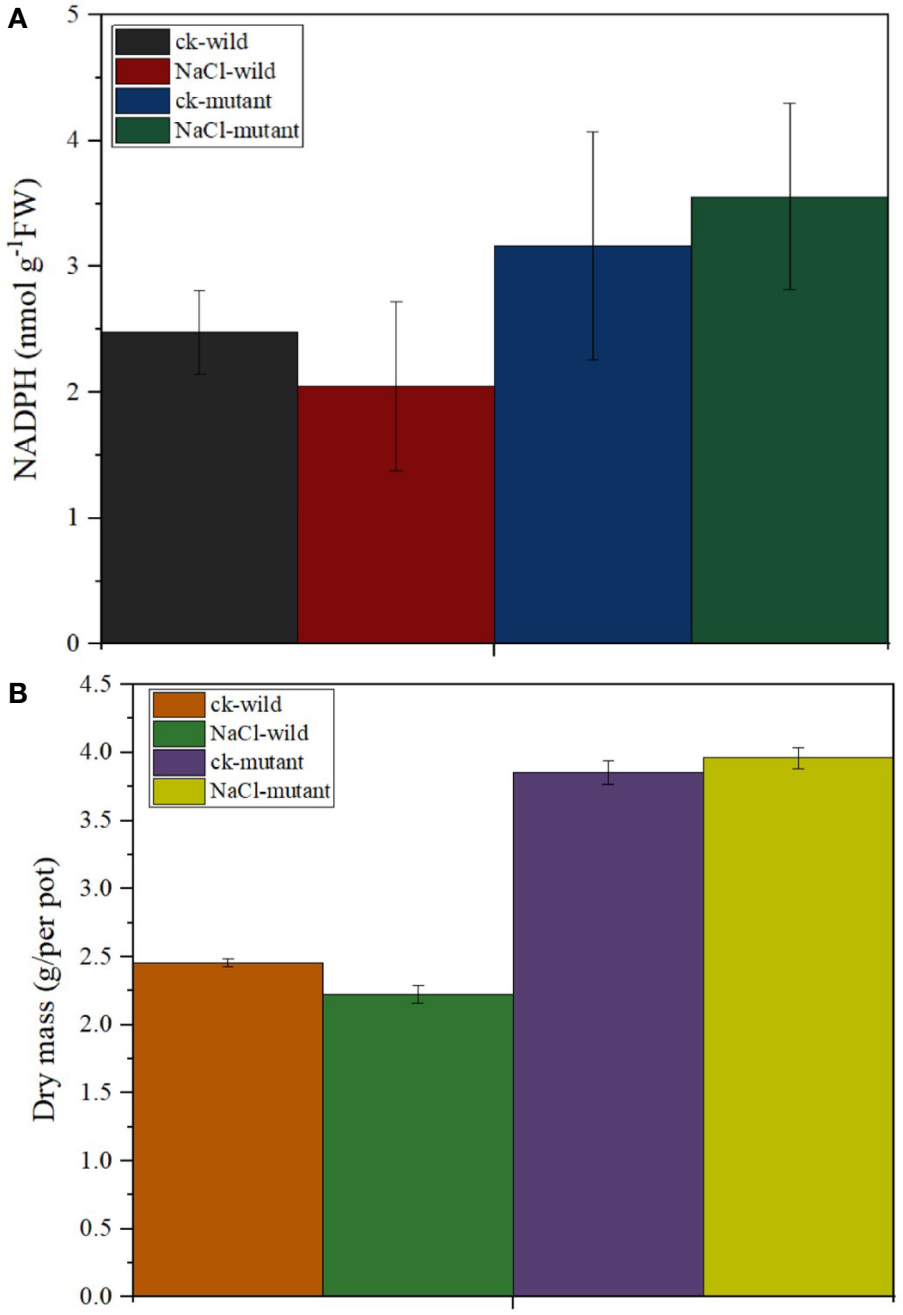
**(A)** Stem-loop secondary structures of MIR171f of bermudagrass. Segments corresponding to the mature miR171f are shown in orange, **(B)** Morphological variation in the control, wild type, and mutant barrelclover plants. NADPH and dry matter accumulation levels.

MiR171f precursor sequence and its promoter were cloned from the C43 bermudagrass precursor sequence and the salt tolerance of transgenic *M. truncatula* was analyzed. The results showed that MiR171f contained an intact stem-loop structure (**Figure S1A**). Overexpression of the MiR171f precursor in *M. truncatula* induced morphological changes both in the shoots and roots. Notable changes included greener leaves and denser canopy compared to the wild as well as the roots as depicted by **Figure S1B**. The mutant under salt stress had the highest accumulation of NADPH followed by the mutant control. While salinity caused a decline in NADPH in the wild compared to the control. However, the decline was not significant (**Figure 9A**). Also, there is a notable increase in dry matter yield in the mutant compared to the wild (**Figure 9B**).

**Figure 10:**
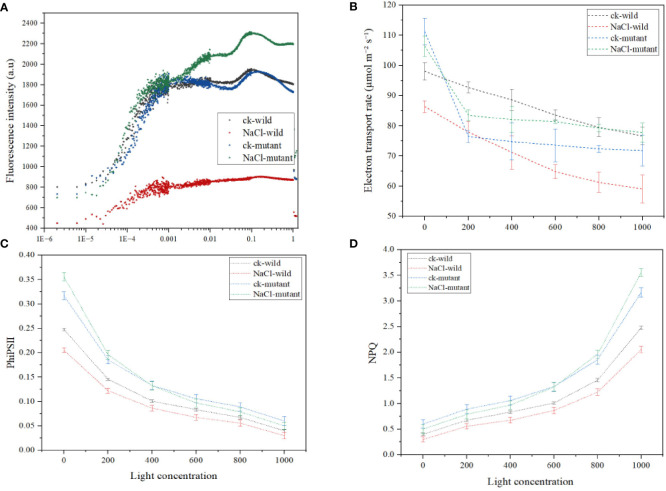
Chlorophyll a fluorescence induction kinetics parameters under salt treatments **(A)** OJIP curve **(B)** electron transport rate **(C)** quantum yield of photosystem II **(D)** nonphotochemical quenching.

The results revealed that different treatments resulted in considerably varied chlorophyll fluorescence behavior. For instance, the wild type of salt treatment had the lowest F_0_ of 441, whereas the control wild had a much higher F_0_ of 820. The F_0_ was medium in the two mutants. Generally, after the first F_0_, all treatments experienced an O-J increase that occurred between 0.00001-0.0001 s. To achieve the greatest F_M_, the J-I-P phase of the fluorescence induction curve rise time spanned from 0.0001-0.001 s. The salt mutant had the highest F_M_ value of 2300 a.u, followed by the wild control, and the wild type of treatment had the lowest of 730.4 au. Notably, the J-I-P rise was much delayed in the wild type of salt regime (**Figure 10A**). The salt regime of the mutant exhibited the sharpest decline in the electron transport rate while the mutant salt treatment had the second highest (**Figure 10B**). The mutant also exhibited the highest initial PhiSII (**Figure 10C**) which despite the drop with the increase in light was the highest among the treatments. A faster rise in NPQ is also observed in the mutant (**Figure 10D**).

**Figure 11:**
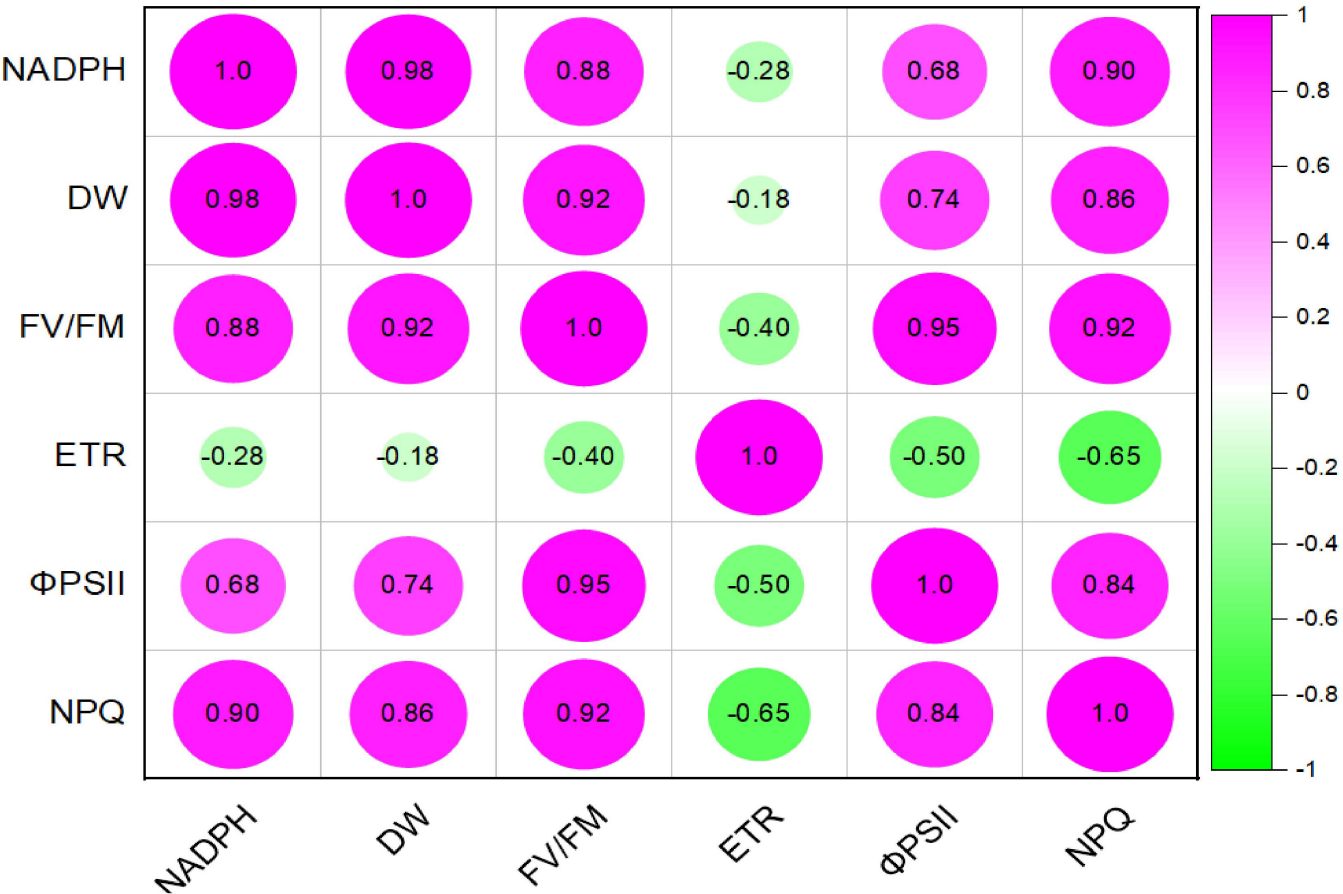
pearssons correlation plot of various light response chlorophyll a fluorence parameters, NADPH and dry matter of mutant *Medicago truncatula* overexpressing bermudagrass miR171f under saline conditions.

Multivariate analysis of the mutant reveals high contribution of NADPH to the dry matter content followed by the F_V_/F_M_. The least correlation is observed in ETR. Other high positive correlation is observed in F_V_/F_M_ and phiPSII, and F_V_/F_M_ vs NPQ (**Figure 11**).

## 4 Discussion

Since photosynthesis is one of the most stress-responsive functional phenotypes, the impact of salinity on the photosynthetic machinery is critical (Hameed et al., 2021). Typically, salinity imposes physiological drought disrupting the balance between foliar transpiration and root water intake (dos Santos et al., 2022). For most crops, the water-stressed leaves harbor 90% of total plant chlorophyll which constitutes the primary light-harvesting complex responsible for triggering the initial photochemical events (Lokstein et al., 2021). Effectively, salinity rapidly disrupts the reduction-oxidation properties of photosystem II acceptors and reduces the photosynthetic electron transport efficiency in both PSI and PSII (Mathur et al., 2014). Thus, attributed to this sensitivity, the genetic responses to stress such as salinity can be very dynamic and complex in nature (Nongpiur et al., 2016). Here, to gain more insight into the mechanism of salt perception and tolerance in bermudagrass, we comparatively analyze how the grass modifies its gene expression post-transcriptionally in photosynthetic leaves.

In addition to their roles in the growth and development and maintenance of genome integrity, miRNAs are important components in plant stress responses (Zhang, 2015). In this study, most of the novel miRNAs detected have a remarkably low abundance (less than 10 reads) indicating that Illumina sequencing was an effective tool in offering a rich source of unreported small RNA data in bermudagrass leaves. The dominance of 22 nt under salt treatment in both regimes suggests that the miRNAs with this length might play more dominant roles in bermudagrass leaf response to salt stress in both cultivars. This is consistent with previous studies in other genera members such as maize (*Z. mays*) (Barber et al., 2012), and barley (*Hordeum vulgare*) (Deng et al., 2015), as well as radish (*Raphanus sativus*) (Sun et al., 2015) and Chinese populus (*Populus tomentosa)* (Ren, 2013) growing under salt stress. Also, most members from conserved families such as miR166, miR156, miR167, miR172, and miR168 had a remarkably high abundance (more than 1000 in the four libraries). This indicates that the overexpression of these miRNAs might be involved in maintaining normal biological functions in the four bermudagrass libraries. Despite a majority having a low abundance, some of the novel miRNAs identified from four libraries had a relatively high abundance (≥10), and their expression levels changed significantly in salt-tolerant regimes. For example, six of the 11 most abundant novel miRNAs are downregulated while only five are upregulated. This similar trend is observed in total miRNAs expressed in C43 vs C198 salt regime whereby most miRNAs are downregulated. These results suggested that the downregulation of not only known but also novel miRNAs play a role in salt tolerance in bermudagrass leaves.

Noteworthy, some of the abundant miRNAs whose targets were not related to photosynthesis from our gene ontology study have been recently confirmed to play vital roles in photosynthesis in other species. For example, suppression of miR166 through gene editing influenced CO_2_ assimilation in rice (Iwamoto, 2022). MiR396 was not only found to be salt responsive in Arabidopsis (Song et al., 2019) and maize (Ding et al., 2009) but also to regulate the expression of chlorophyll biosynthetic genes (Wang et al., 2020). Pan et al. (2016) observed that miR172 improves photosynthetic performance in soybean. Furthermore, alfalfa genotypes overexpressing miR156 exhibited altered photosynthesis activity under salinity conditions (Arshad et al., 2017). Also, it has been shown that rice mutants with suppressed miR390 exhibited an increase in chlorophyll accumulation (Ding et al., 2016). For miRNAs whose targets are in the photosynthesis pathway in this study, 4 are conserved (miR171f, miR319, miR156, and miR159) while 3 are nonconserved. Interestingly, the targets fall in the electron transport photosynthesis and NAD^+^ binding. In a closely related species to bermudagrass, it was reported that the overexpression of miR319 in creeping bentgrass significantly enhanced tolerance to salt stress by enhancing the photosynthetic performance. Generally, these observations indicate that the downregulation of these miRNAs in the C43 vs C198 salt regime demonstrates a possible regulatory role in photosynthetic regulation through transcriptional repression under salt stress.

Generally, miRNA activity leads to the repression of genes and transcription factors they target. Therefore, the upregulation of miR171f under saline conditions in salt tolerant variety demonstrates that the suppression of gene targets is a prerequisite for desirable phenotypes. miR171f targets the *Pentatricopeptide repeat-containing protein At3g62470* gene located in the chloroplasts which are associated with electron transporter, transferring electrons within the cyclic electron transport pathway of photosynthesis activity. This is consistent with the fact that light-dependent reactions of photosynthesis take place in the thylakoid membrane, inside chloroplasts (Merchant and Sawaya, 2005). It is also in the chloroplasts where the light-harvesting complex is located. Here, chlorophyll fluorescence occurs transforming light energy into chemical energy (Papageorgiou, 2004). In this study, following the initial F_0_, all treatments saw an O-J rise suggesting that salinity modifications had no effect on this phase. This phase represents the photochemical step of Chl fluorescence induction. Thus, higher F_0_ values in the mutant relative to the wild type showed a larger physical separation of the PSII reaction center from their corresponding pigment antennae, which has been shown to contribute to better photosynthetic performance by restricting energy input into the electron transport chain (Strasser et al., 2000). The J-I-P phase of the fluorescence induction curve rising time was set at 0.0001-0.001 s to produce the highest F_M_. Notably, the wild-type J-I-P increase was substantially delayed, demonstrating plastoquinone accumulation, whereas it increased in the mutant. The large increase in I translates to slower electron transit to the PSI acceptors (Joliot and Johnson, 2011). In mutants, there is also a higher plateau, indicating a bigger number of PSI end acceptors, which are typically linked with alternative electron transfer routes that function as electron sinks (Popova et al., 2022). During the photochemical reactions, plants use water to make NADPH which is then metabolized to release electrons (Wasilewska-Dębowska et al., 2022). the NPQ influences overall plant photosynthesis, biomass, and yield by preventing photoinhibition and temporarily limiting photosynthetic quantum output. (Murchie and Ruban, 2020). In this study, a positive correlation between NPQ and F_V_/F_M_ is observed from the boot stage indicating that most light dissipation occurred at this stage while at flowering some varieties had begun to exhibit photoinhibition. Further, the NPQ operation is related to the state of reduction of the thylakoid electron transport (Alboresi et al., 2019). This is an important point because the level of protection required depends on the balance between the amount of absorbed irradiance and the ability of the system to use this energy within the various ‘sinks’ for electrons (Joliot and Johnson, 2011). Thus, it is important to determine the rate of flow of electrons. The NADPH is a product of the first level of photosynthesis (Johnson, 2017). It helps to fuel the reactions that occur in the second stage of the process of photosynthesis (Lim et al., 2020). Higher production of NADPH in mutants indicates that miR171f promotes higher electron donation under salt stress. The released electrons must travel through the photosystem II protein and down the electron transport chain then they pass through photosystem I (Cooper, 2000). Thus, higher accumulation of PhiPSII therefore indicated high efficiency in the transfer of electrons in the mutants that the wild type.

**Figure 12:**
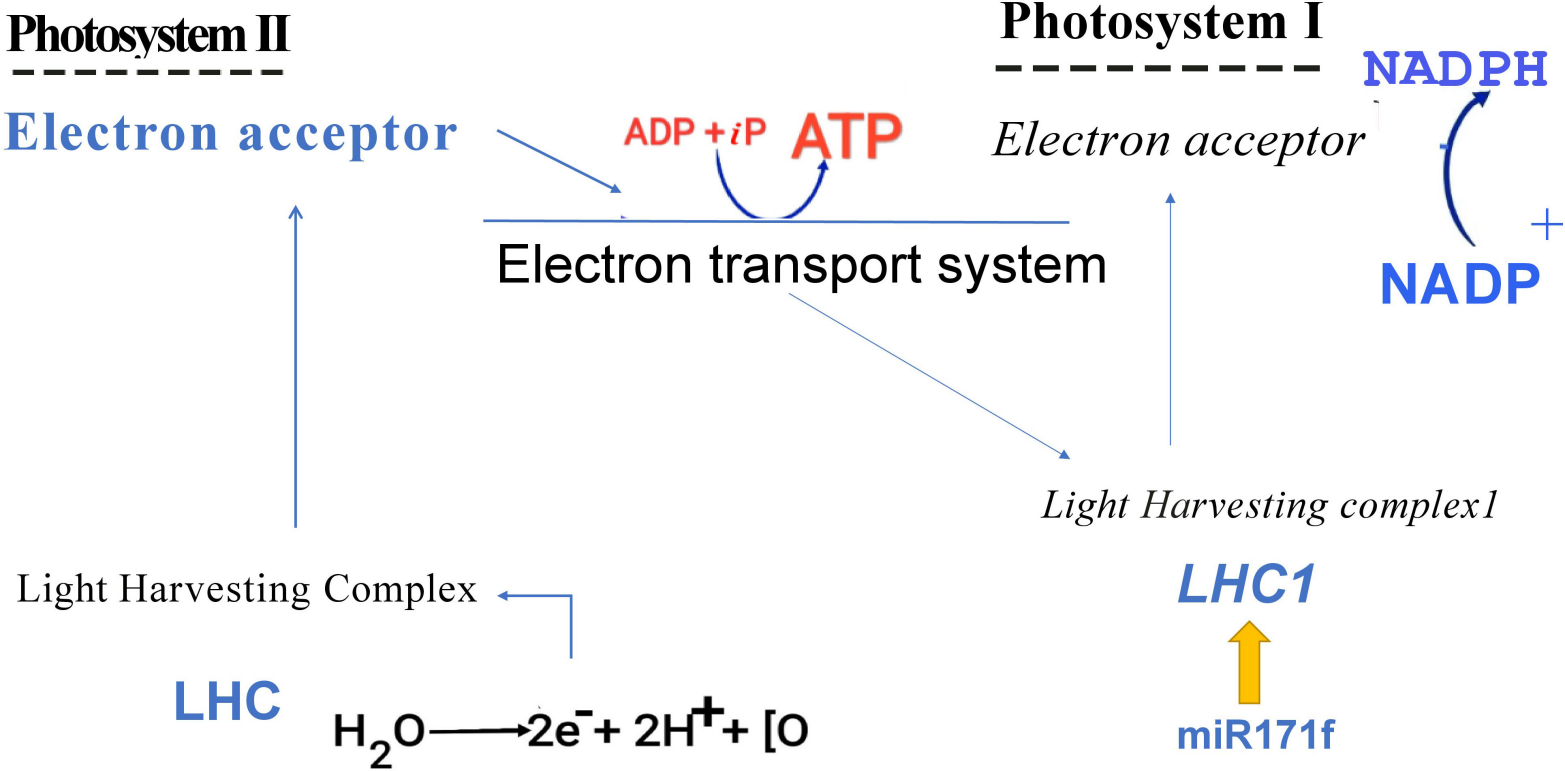
Graphical representation of proposed mechanism.

Chlorophyll constitutes the primary light-harvesting complex responsible for triggering the initial photochemical events (Lokstein et al., 2021). Here, we proposed that miR171f mediated transcriptional regulation of LHC1 modulates the reduction-oxidation properties of photosystem II acceptors and enhances the photosynthetic electron transport efficiency in both PSI and PSII (Mathur et al., 2014) which influences the NADPH synthesis from NADP (**Figure 12**).

## 5 Conclusions

In conclusion, we used RNA sequencing to profile miRNAs in two bermudagrass cultivars with variable degrees of salt tolerance when subjected to salt stress. The bulk of the 536 salt-inducible miRNA variations were downregulated in salt-tolerant cultivars. Seven miRNAs targeted six genes associated with electron transport, dark reaction photosynthesis, and NAD+ binding. MiR171f targeting a Pentatricopeptide repeat-containing protein *Cd3g62470* and Aldehyde dehydrogenase family 3 member F1, both of which are considerably enriched in the electron transport route of light response photosynthesis, were dramatically increased in salt tolerant regimes. To enable genetic breeding for photosynthetic capacity, we overexpressed miR171f in *Medicago truncatula*, which led in a considerable improvement in photosynthetic performance and biomass accumulation in the mutants under saline circumstances compared to the wild type. While not very qualitative, these findings provide significant insights into plant breeding.

## Data availability statement

The data presented in the study are deposited in the NCBI repository, accession number PRJNA287735.

## Author contributions

SF performed the statistical analysis and wrote the first draft of the manuscript, EA conducted the research, SA wrote sections of the manuscript, YL and YY contributed to conception and design of the study. All authors contributed to the article and approved the submitted version.
