# Supplementary figures and images for: Salt-responsive bermudagrass microRNAs and insights into light reaction photosynthetic performance
Source: Front Plant Sci. 2023 Feb 15;14:1141295. doi: 10.3389/fpls.2023.1141295 (PMC9975589; doi:10.3389/fpls.2023.1141295)

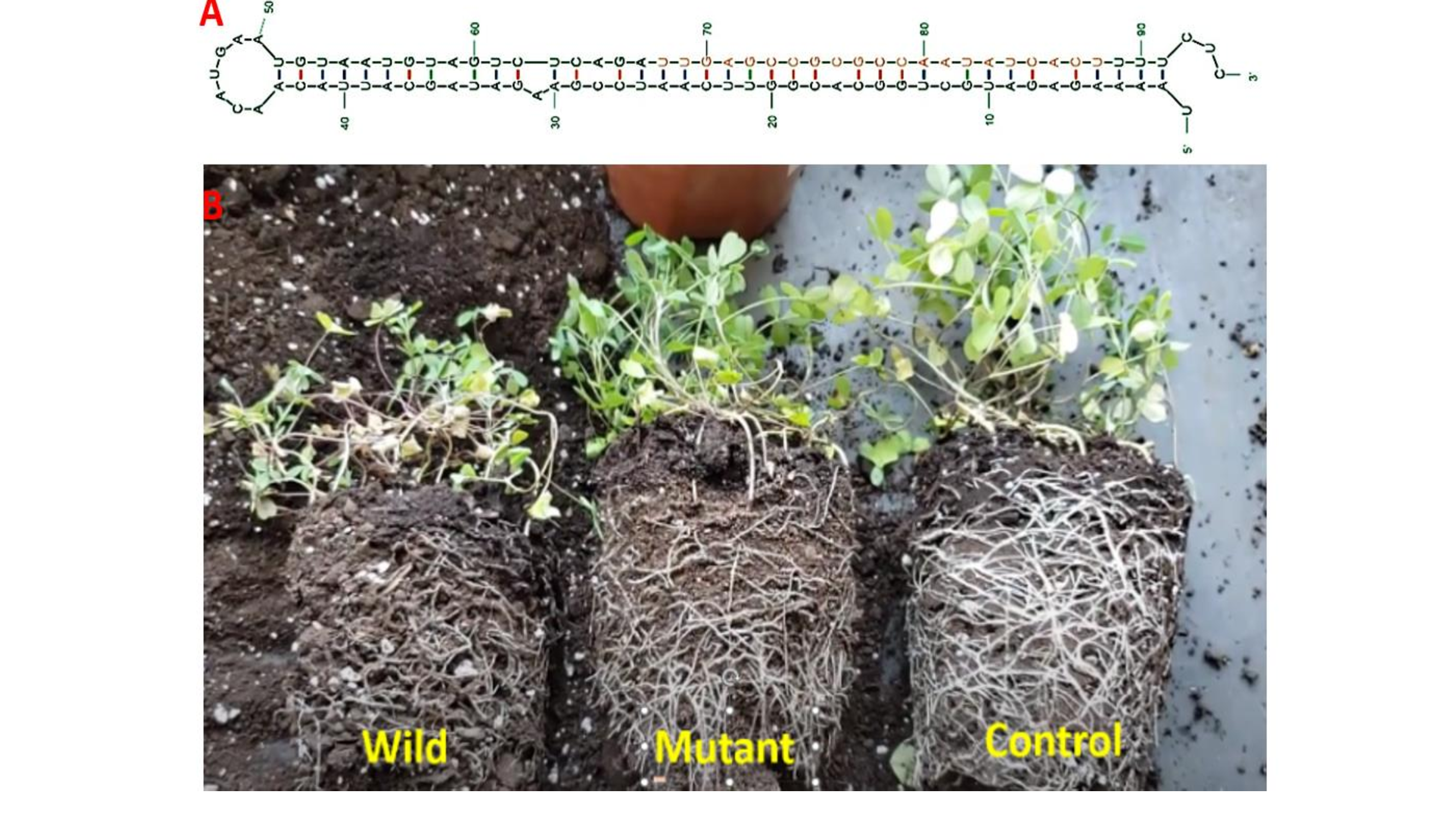

Supplement: Supplementary file 1 [file Image_1.png]
